# Biotic interactions between the human pathogen *Legionella pneumophila* and nematode grazers in cooling tower biofilms

**DOI:** 10.1371/journal.pone.0309820

**Published:** 2024-10-25

**Authors:** Christin Ortlieb, Aurélie Labrosse, Liliane Ruess, Michael Steinert

**Affiliations:** 1 Institute of Biology, Ecology, Humboldt-Universität zu Berlin, Berlin, Germany; 2 Institute of Microbiology, Technische Universität Braunschweig, Braunschweig, Germany; Maria Curie-Sklodowska University, POLAND

## Abstract

Biofilms in cooling towers represent a common habitat for the human pathogen *Legionella pneumophila*. Within the biofilm consortium, frequent interactions with protozoa, i.e. amoebae and ciliates, were reported, while nematodes have only recently been considered as potential environmental reservoir for the pathogenic bacteria. This study is the first approach to investigate the biotic interactions between *L*. *pneumophila* and bacterial-feeding nematodes in a semi-natural biofilm model. The species were *Diploscapter coronatus*, *Diploscapter pachys*, *Plectus similis* and *Plectus* sp., which all co-occur with *L*. *pneumophila* in the environment. Biofilms derived from cooling towers were either inoculated with mCherry-labeled *L*. *pneumophila* solely or in combination with GFP-labeled *Escherichia coli*. All experiments were conducted in single-species set-ups and multi-species (*D*. *coronatus* and *P*. *similis*) set-ups, to account for interspecific competition. Bacterial ingestion was assessed after 24 and 96 h as fluorescence patterns in the digestive tract of the nematodes using confocal laser scanning microscopy. *L*. *pneumophila* cells were ingested by all nematode species, with *D*. *coronatus* having the highest pathogen load. The fluorescence intensity (i.e. bacterial load) varied between compartments within the digestive tract and was independent of incubation time. Bacterial cells accumulated mostly around the cardia and in the intestine, while less cells were found within stoma and pharynx. Interspecific competition changed the pattern, i.e. with incubation of *D*. *coronatus* and *P*. *similis* in the same biofilm a significantly higher pathogen load occurred in the intestine of *D*. *coronatus* than *P*. *similis* after 24 h and 96 h. Remarkably, when given a choice between *L*. *pneumophila* and *E*. *coli*, *P*. *similis* was the only nematode species containing both bacteria after incubation for 24 h. None of the other nematode species contained *E*. *coli* after 24 h and 96 h incubation, while *L*. *pneumophila* was present. This study thus provides the first evidence, that under environmental conditions *L*. *pneumophila* is a frequent diet of bacterial-feeding nematodes, highlighting their potential as pathogen vectors or even host in cooling tower habitats.

## 1. Introduction

In the aquatic environment, prokaryotic and eukaryotic cells often join together to form complex communities adhering to inorganic or organic surfaces, known as biofilms [1]. Here, cells are embedded in a matrix of extracellular polymeric substances (EPS), which protects from a wide range of environmental challenges, e.g. predation, UV exposure, metal toxicity, dehydration and salinity, phagocytosis as well as antimicrobial agents [2, 3]. Especially in man-made water systems (e.g. dental units, drinking water systems, showerheads, air conditioning units, cooling towers), the enhanced resistance of biofilms to antibiotics is a serious concern for human health [4–7]. One bacterial pathogen that naturally grows and persists in such technical water systems is *Legionella pneumophila*, which is also classified as one of the highest-priority infectious disease pathogens of general public health concerns in Germany [8, 9].

*L*. *pneumophila* is the causative agent of a severe pneumonia called Legionnaires’ disease, with a case fatality rate of 9% in Europe [10]. The inhalation of *Legionella*-contaminated aerosols can result in a bacterial infection of the human lungs, leading to alveolar damage, cellular infiltrations of neutrophils and macrophages, and eventual dispersal of extracellular bacteria to other organs [11]. Large community-associated outbreaks of Legionnaire’s disease are most frequently associated with cooling towers, which can disperse *Legionella*-contaminated aerosols over long distances of up to 10–15 km [12, 13]. In Germany, at least four cooling tower-related outbreaks of Legionnaires’ disease were documented since 2009 [14]. The largest outbreak to date with 159 suspected cases occurred in 2013 in Warstein [15].

Cooling towers provide an excellent environment for the growth and proliferation of diverse microorganisms due to elevated operation temperatures, a neutral pH and continuous aeration [16]. Within cooling towers, the availability of surfaces in the evaporative fill material, heat exchangers, water reservoir and cooling water pipelines allow for the extensive growth of biofilms [17]. Particularly the water temperatures between 25°C and 35°C support the growth of the thermophilic *L*. *pneumophila* [18]. The majority of cooling towers apparently contain a core *Legionella* community, that quickly recovers after chemical disinfection [19]. Especially the floating biofilms at the water-air interface in the basin represent a common bacterial habitat [20].

As an intracellular parasite *L*. *pneumophila* is regularly found in microbial grazers, predominantly amoebae (e.g. *Acanthamoeba*, *Vermamoeba*, *Naegleria*) and ciliates (*Tetrahymena*) [21]. Amoebae isolated from cooling towers are 16 times more likely to be infected (albeit not necessarily with *Legionella*) than amoebae isolated from natural fresh waters [22], making cooling towers a hot spot for *Legionella*-amoebae interactions [23]. However, virtually nothing is known on the interactions with other major bacterial grazers, the nematodes, in the biofilms of cooling towers.

Among metazoa, nematodes are the most abundant and diverse group in both natural biofilms and granular or biological filters in technical waters [24, 25]. As filter-feeders, nematodes ingest bacteria suspended in liquid. Food particles are transported from the stoma to the intestine via the pharynx, a neuromuscular organ functioning as pump [26]. A grinder located in a bulb at the end of the pharynx crushes the bacteria before they are pushed through a valve and enter the intestine [27]. By grazing on bacteria nematodes influence key biofilm processes such as detachment, oxygen turnover, carbon mineralization and release of secondary metabolites [28, 29]. Nematode feces and mucus excreta may further enhance microbial growth, while bioturbation increases biofilm permeability and provides new niches for bacteria [30, 31].

Generally, *L*. *pneumophila* cells can survive the passage through the nematode grinder and colonize the intestinal tract [32]. Here, the pathogen replicates in the intestinal lumen but also invades intestinal cells establishing *Legionella*-containing vacuoles (LCVs) [32, 33]. Colonization and persistence of the pathogen within the intestine leads to a shortened lifespan compared to individuals fed with *E*. *coli* OP50, as shown for *C*. *elegans* [32]. Next to this parasitic relationship, host-bacteria interactions can be commensal, i.e. bacteria obtain nutrients off its host without introducing disease [34].

By harboring and excreting bacteria in viable conditions, nematodes contribute to their dissemination in the environment, which makes them a potential health risk as vectors of human pathogens including *Legionella*. However, infection assays with nematodes are mainly restricted to the model host *Caenorhabditis elegans* [32, 33, 35], which is typically not present in aquatic environments. Recently, a survey screening the biofilms of natural (26 swimming lakes) and technical (7 cooling towers) water habitats, revealed that nematodes can act as potential reservoirs, vectors or grazers of *L*. *pneumophila* in cooling towers [36]. Seven nematode taxa that thrive in *Legionella*-positive cooling tower biofilms were isolated and cultured, including *Plectus* and *Diploscapter*. This provides an ideal basis for studying the interactions between nematodes and *Legionella* with biota that co-occur in nature, to address the role of nematodes as environmental reservoirs. In a previous study, we were able to show that the *L*. *pneumophila* cooling tower isolate KV02 impairs the feeding activity of the environmental biofilm isolates *P*. *similis* and *Plectus* sp. [36]. Examination of the pharyngeal pumping activity showed, that *L*. *pneumophila* decreased the pumping rate of both nematode species in comparison to the standard lab food *E*. *coli* OP50 by 70% [36]. This suggests that the pathogen, at least in an artificial set-up on an agar plate, is not a common food source for nematodes.

In order to further decipher the *Legionella*-nematode interaction, the current study investigates the relationship between *L*. *pneumophila* and bacterial-feeding nematodes in an ecological set-up, i.e. a semi-natural biofilm model. To achieve the most realistic picture of this interspecific interaction, the tested *L*. *pneumophila* strain KV02, the nematode *P*. *similis*, and the biofilm material were all isolated from the same cooling tower. Additionally, *Plectus* sp., isolated from a natural *Legionella*-positive spa bath [37], was used. Further, the thermophilic *Diploscapter coronatus* and *Diploscapter pachys* were tested. *Diploscapter* is a common taxon in cooling tower biofilms and known vector for enteric pathogens such as *Salmonella* and *Listeria* [36, 38]. The taxa *Plectus* and *Diploscapter* have different ecological life-strategies. *Diploscapter* is an enrichment opportunist, continuously pumping with its pharynx at high microbial densities, while *Plectus* is a generalist opportunist, pumping less frequently and also surviving on poor food conditions [39]. These different feeding strategies of the two tested nematode taxa may affect the grazing activity on *L*. *pneumophila*. Test assays were performed with *Legionella* only and *Legionella* with the common food bacterium *Escherichia coli* in combination to investigate the food choice of nematodes. *L*. *pneumophila* was labeled with mCherry, and *E*. *coli* with GFP, to allow tracing of cells in biofilm and nematodes. The following hypotheses were investigated (1) Ingestion of *L*. *pneumophila* by nematodes varies between species, with enrichment opportunists showing the highest uptake, (2) the distribution pattern of bacterial cells within the digestive tract of nematode grazers differs due to interspecific interactions (e.g. commensalism or parasitism), and (3) nematodes prefer *E*. *coli* over the pathogen *L*. *pneumophila* when given a choice.

## 2. Materials and methods

### 2.1 Cultivation of bacterial strains

The *Escherichia coli* strain DH10β harboring the GFP-expressing plasmid pXDC31 was cultivated on lysogeny broth (LB) agar supplemented with 12.5 μl ml^-1^ chloramphenicol. The *E*. *coli* strain OP50 was grown in LB medium. Both *E*. *coli* strains were long-term laboratory cultures and were incubated on the respective media overnight at 37°C. The *L*. *pneumophila* strain KV02 was isolated from biofilms of a cooling tower [36]. The bacteria were labeled with mCherry (plasmid pXDC50) and grown on buffered charcoal-yeast extract (BCYE) agar, supplemented with 0.5 mM isopropyl-β-D-thiogalactopyranoside (IPTG) and 5 μl ml^-1^ chloramphenicol at 37°C for 2 days. Prior to the experiments, bacteria were carefully removed from the agar plate and resuspended in sterile-filtered cooling tower water. Then, bacterial density, expressed as colony forming units (CFU ml^-1^), was measured at OD600nm.

### 2.2 Cultivation of nematodes

All assays were performed with free-living, bacterial-feeding nematodes, reported as common fauna in cooling tower biofilms [36]. This comprised the generalist opportunists *Plectus similis* (0.53 ± 0.10 mm; Zell 1993) and *Plectus* sp. (0.44 ± 0.80 mm), and the enrichment opportunists *Diploscapter coronatus* (0.45 ± 0.63 mm; Cobb 1893) and *Diploscapter pachys* (0.38 ± 0.61 mm; Steiner 1942). The taxa Diploscapter (c-p 1) and *Plectus* (c-p 2) are grouped along a colonizer-persister continuum ranging from one to five on the basis of several character sets, including the reaction to resource pulses [40].

*P*. *similis* was isolated from a cooling tower biofilm in Lower Saxony, Germany [36] and *Plectus* sp. from a biofilm in a thermal spa bath in Aix-les Bains, France [37]. Both *Plectus* species were grown on Page’s Amoeba saline (PAS) agar on their natural bacterial microbiome, without additional food supply. *D*. *coronatus* was kindly provided by Dr. Philipp Schiffer and the *D*. *pachys* strain PF1309 by Hélène Fradin [41]. Stock cultures of *Diploscapter* were grown on nematode growth medium (NGM) agar plates inoculated with an *E*. *coli* OP50 lawn at 20°C. Since nematodes do not fall under the animal welfare regulations in Germany (i.e. laboratory animal reporting regulations), no Ethics statement is required. The same applies to the flora and fauna originating from the biofilm material.

### 2.3 Biofilm assay

Natural biofilms were obtained from a cooling tower with proven occurrence of *L*. *pneumophila* located in the Lower Saxony region in Germany [36]. Floating biofilms from the air-water interface were collected in glass bottles and stored at 4°C until further processing. The cooling tower biofilm was mainly composed of benthic cyanobacteria (predominantly *Lyngbya*, *Oscillatoria*, *Phormidium*), benthic Charophyta (predominantly *Klebsormidium*) and epiphytic Bacillariophyta (predominantly *Amphora*, *Navicula*, *Nitzchia*).

To achieve nematode-free biofilms, 20 ml subsamples were screened for biofilm-dwelling nematodes using an inverse microscope (CKX31, Olympus, Tokyo, Japan). To avoid food competition between the autochthone grazers and the four test nematode species, the natural nematode fauna was removed using a micropipette. The removal of nematode eggs was not possible, yet the feeding pressure of newly hatched nematode larvae can be considered as minor. Then, a volume of each 250 μl nematode-free biofilm were transferred into chamber slides (each chamber with a total volume of 300 μl; ibidi, Gräfelfing, Germany) as experimental microcosms (S1 Fig).

For the inoculation of natural biofilms with bacteria and nematodes we adapted a method described by Rasch et al. [37]. In the *L*. *pneumophila*-only assay, the feeding of nematodes on the pathogenic bacteria inhabiting the biofilm was tested. For this, 50 μl of mCherry-labeled *L*. *pneumophila* KV02 (final concentration 2 x 10^8^ CFU ml^-1^), resuspended in sterile-filtered cooling tower water, were inoculated and left overnight at 25°C to adapt. As next step, hand-picked test nematodes (30 individuals per microcosm) were inoculated per microcosm (n = 2). Nematodes were age-synchronized by selecting only young females (visual inspection using a binocular) in order to exclude age-dependent differences in ingestion rates. Two scenarios were investigated: *i)* single set-ups with one of the four test species each, and *ii)* mixed set-ups with a combination of *P*. *similis* and *D*. *coronatus* with each 15 individuals per microcosm (n = 2). These two species were chosen because they are most likely to share technical biofilms as habitat: *P*. *similis* occurred in cooling tower biofilms together with *L*. *pneumophila* [36] and *D*. *coronatus* was abundant in biofilter reactors [42].

For the second test series, the *L*. *pneumophila* versus *E*. *coli* assay, 50 μl of GFP-labeled *E*. *coli* DH10β were inoculated together with 50 μl of mCherry-labeled *L*. *pneumophila* KV02 (final concentration 6 x 10^8^ CFU ml^-1^) prior to the addition of nematodes. The same two scenarios as for the *L*. *pneumophila*-only assay were tested, either single set-ups (one of the four test species with 30 individuals per microcosm) and mixed set ups (combination of *P*. *similis* and *D*. *coronatus* with 15 individuals each) per microcosm (n = 2). All microcosms were incubated at 25°C for either 24 h or 96 h. This time span covers the initial ingestion of bacteria until reaching a plateau of the intestinal bacterial load [32, 43]. Prior to killing the nematodes with 15% paraformaldehyde for microscopic analysis, 50% of the individuals were checked for viability using a binocular microscope.

The palatability of GFP-labeled *E*. *coli* DH10β for all nematode species was verified in preliminary choice tests after Abada et al. [44] against the isogenic strain DH10β not expressing GFP and against *E*. *coli* OP50 (S2 Fig). Both, *E*. *coli* DH10β and *E*. *coli* OP50 are common lab foods of nematodes [45, 46]. Yet, as *D*. *coronatus* and *D*. *pachys* were grown on *E*. *coli* OP50 the strain *E*. *coli* DH10β was used for the biofilm assays to minimize potential effects of food familiarity. Additionally, ingestion of GFP-labeled *E*. *coli* DH10β in sterile-filtered cooling tower water was confirmed microscopically (S3 Fig).

### 2.4 Fluorescence microscopy and data analysis

The biofilm microcosms were screened for nematode colonization and ingestion of *L*. *pneumophila* KV02 and *E*. *coli* DH10β by confocal laser scanning microscopy (CLSM; Leica TCS SP8, Wetzlar, Germany). The excitation wavelengths 488 nm and 552 nm were used to excite GFP and mCherry, respectively. Emission spectra were collected between 492 and 544 nm (GFP) and 564 and 724 nm (mCherry). In some cases, full z-series images were obtained for 3D animations to verify the rod shape of single bacterial cells and to verify that the bacterial cells were located within the nematode body and not outside on the cuticula. To account for nematode autofluorescence, potentially interpreted as bacteria-derived fluorescence, nematodes from microcosms without labeled bacteria served as control.

The mean fluorescence intensity (MFI) of mCherry-labeled *L*. *pneumophila* KV02 and GFP-labeled *E*. *coli* DH10β was quantified in the nematode digestive system. The software Fiji was used, an open-source platform focused on biological-image analysis [47]. The final MFI (bacterial signal) was calculated by subtracting the MFI of the non-fluorescent image background from that of the body tissue MFI. The MFI data are given as arbitrary unit (a.u.) ± standard error (SEM). Graphs are expressed as mean per nematode, with nematode individuals as biological repeats composed of 2 technical replicates (i.e. 2 microcosms).

**Fig 1:**
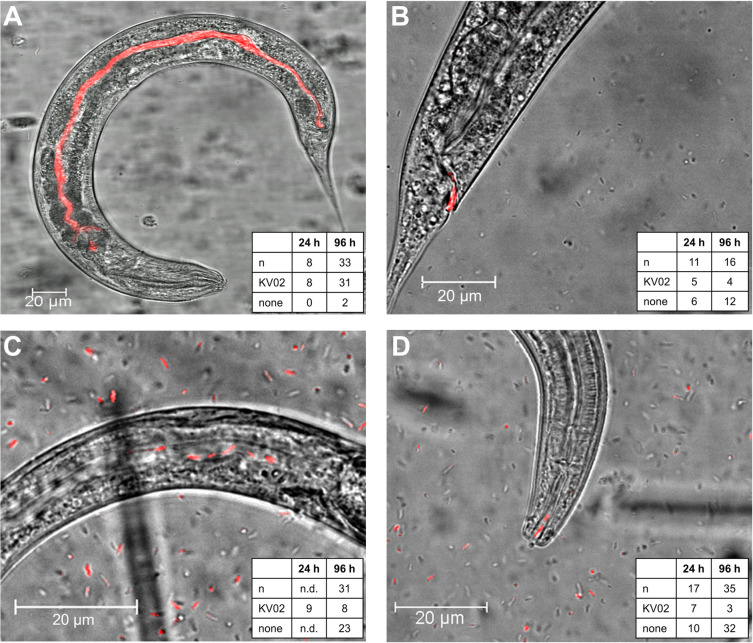
Representative confocal laser scanning microscopy images of nematodes harboring *Legionella pneumophila* KV02. (A) *Diploscapter coronatus*. (B) *Diploscapter pachys*. (C) *Plectus similis*. (D) *Plectus* sp. Nematodes harbor *L*. *pneumophila* KV02 (mCherry, red) in the stoma (*Plectus* sp.), terminal bulb (*D*. *coronatus*), cardia (*D*. *coronatus*), intestine (*D*. *coronatus*, *P*. *similis*), and rectum (*D*. *coronatus*, *D*. *pachys*). Number of investigated individuals (n), number of individuals that harbored *L*. *pneumophila* KV02 (KV02) and number of individuals without *L*. *pneumophila* KV02 (none) after incubation for 24 h and 96 h are shown for each nematode species.

The presence of bacteria was assigned in six parts of the nematode digestive tract: mouth cavity (stoma), the oesophagus (pharynx, terminal bulb, cardia), and the gut (intestine, rectum) (Fig 1 and S2 Fig). Each confocal image was scanned for the presence of fluorescent bacteria within nematodes. Then, the entire body part (region of interest, ROI) was circled with a drawing pen and the MFI was measured for the defined area by the software. This procedure was repeated for all nematode individuals with proven ingestion of bacteria and set-up. For each nematode examined the MFI was measured for all six body parts. To ensure that the original image data from the microscope software were comparable, the underlying image properties (e.g. contrast, saturation) were not changed between recordings. Nematodes in which no bacteria were found were not considered for the calculation of the MFI.

The data are analyzed using R version 4. 2. 3 “Shortstop Beagle” [48]. Prior to statistical tests all data were checked for normal distribution and homogeneity of variances. Then, the data were subjected to the Mann-Whitney U test or the Kruskal-Wallis rank sum test with Dunn’s post hoc test (with Bonferroni correction). A significance level of p < 0.05 was applied to all statistical analyses.

## 3. Results

### 3.1 No-choice assays—*L*. *pneumophila*-only

#### Single-species set-up

**Fig 2:**
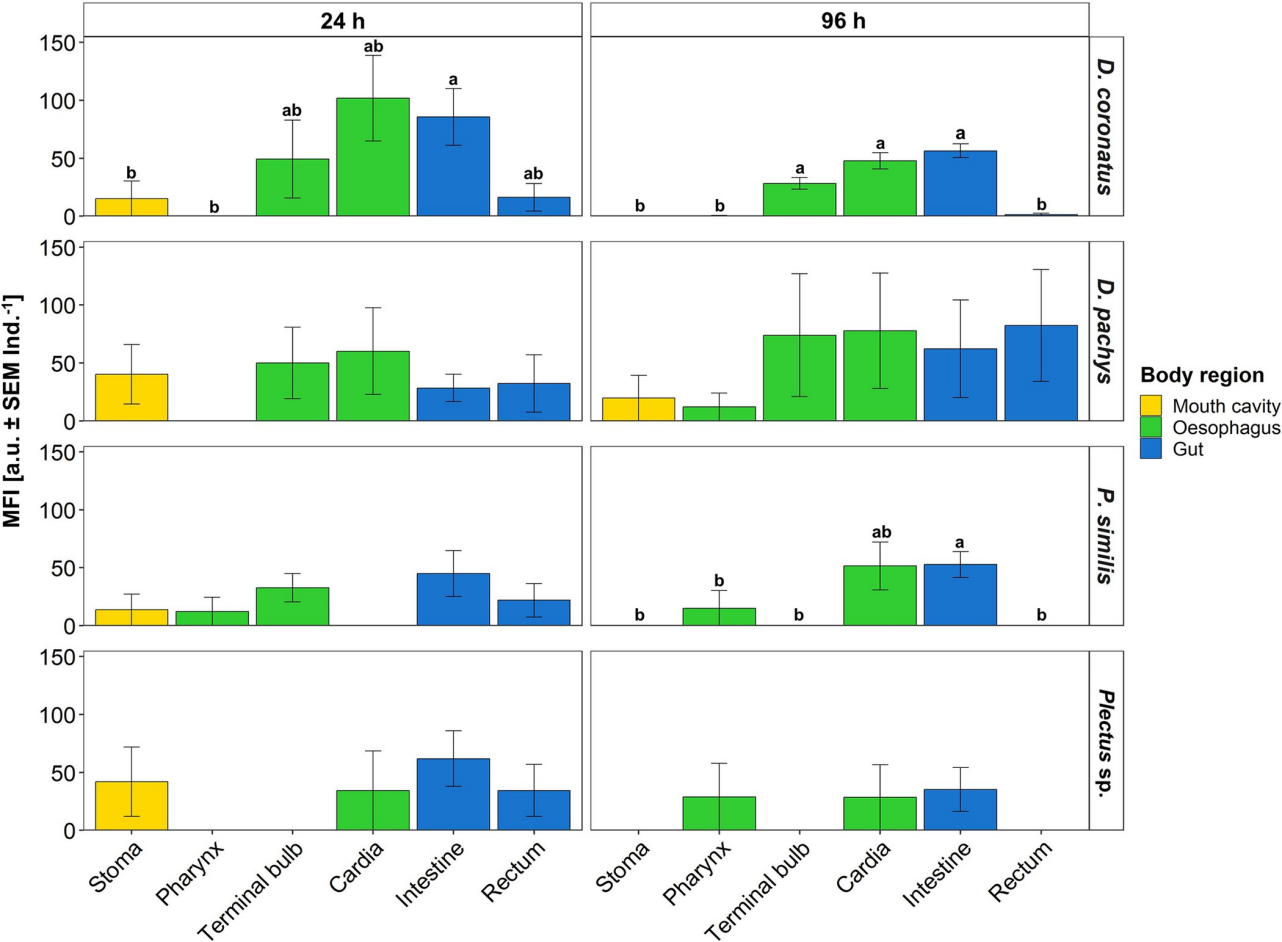
Bacterial load of the digestive system of nematodes after incubation with *Legionella pneumophila* KV02. Bacterial load as measured by mean fluorescence intensity (MFI: mean ± SEM Ind.^-1^). Nematodes were incubated with *L*. *pneumophila* KV02 (mCherry, red) for 24 h and 96 h. Compared were the mouth cavity (stoma), the oesophagus (pharynx, terminal bulb, cardia), and the gut (intestine, rectum). Bars with no or the same letters are not statistically different according to Dunn’s post hoc test (p < 0.05).

With *L*. *pneumophila* KV02 and nematodes in single test assays, bacterial cells were ingested by all nematode species, yet detected at different compartments within the digestive system (Figs 1 and 2). After 24 h incubation all individuals of *D*. *coronatus* harbored bacteria (Fig 1A), with the bacterial load (expressed as MFI) highest around the cardia, followed by the intestine and the terminal bulb, with 102, 86, and 49 a.u. Ind^-1^; respectively (Fig 2). On the other hand, stoma and rectum harbored only few bacteria, which was significant for the stoma compared to the intestine (p < 0.028). After 96 h incubation, the overall MFI decreased by about half, while the fluorescence location pattern remained similar: The bacterial load was again highest in the intestine (56 a.u. Ind^-1^) and around the cardia (48 a.u. Ind^-1^), followed by the terminal bulb (28 a.u. Ind^-1^). The lowest MFI was emitted by the pharynx and rectum (p < 0.001) and no bacteria were detected in the stoma.

After 24 h incubation, *L*. *pneumophila* KV02 was present in 50% of *D*. *pachys* specimen (Fig 1B). The MFI was highest around cardia (60 a.u. Ind^-1^) and terminal bulb (50 a.u. Ind^-1^; Fig 2), and lowest in the intestine (28 a.u. Ind^-1^). As for *D*. *coronatus*, no fluorescence was detected in the pharynx. After 96 h incubation, only 25% of *D*. *pachys* individuals harbored *L*. *pneumophila* KV02. The bacterial load was comparable in terminal bulb, cardia, intestine and rectum, with a mean MFI of 74 a.u. Ind^-1^, but much lower in stoma and pharynx (Fig 2).

After 24 h incubation, *P*. *similis* comprised most bacteria in the intestine and terminal bulb with 45 and 33 a.u. Ind^-1^; respectively (Fig 2). No fluorescence signal was detected around the cardia, while here the MFI was 51 a.u. Ind^-1^ after 96 h. Generally, *L*. *pneumophila* was only found in 26% of individuals after 96 h incubation. The bacterial load was significantly higher in the intestine than in the pharynx (p < 0.049), whereas no bacteria were present in the stoma, terminal bulb and rectum.

After 24 h incubation, *Plectus* sp. harbored *L*. *pneumophila* in 40% of individuals (Figs 1D and 2). The bacterial load was highest in the intestine (62 a.u. Ind^-1^). Otherwise, *L*. *pneumophila* KV02 was distributed evenly between stoma, cardia and rectum, with an MFI of about 37 a.u. Ind^-1^. After 96 h incubation, *L*. *pneumophila* KV02 was only present in 9% of nematodes. The bacteria homogenously spread in the pharynx, cardia and intestine, with an MFI of around 31 a.u. Ind^-1^. Comparable to *P*. *similis*, no fluorescence was detected in the stoma, terminal bulb and rectum.

For each of the four nematode species, the MFI of *L*. *pneumophila* KV02 in the different parts of the digestive system was independent of incubation time (p *>* 0.057). Moreover, the bacterial load of the investigated compartments did not differ between species, except for a significantly higher bacterial load in the rectum of *D*. *coronatus* compared to *D*. *pachys* after 96 h incubation (p < 0.002).

#### Mixed-species set-up

**Fig 3:**
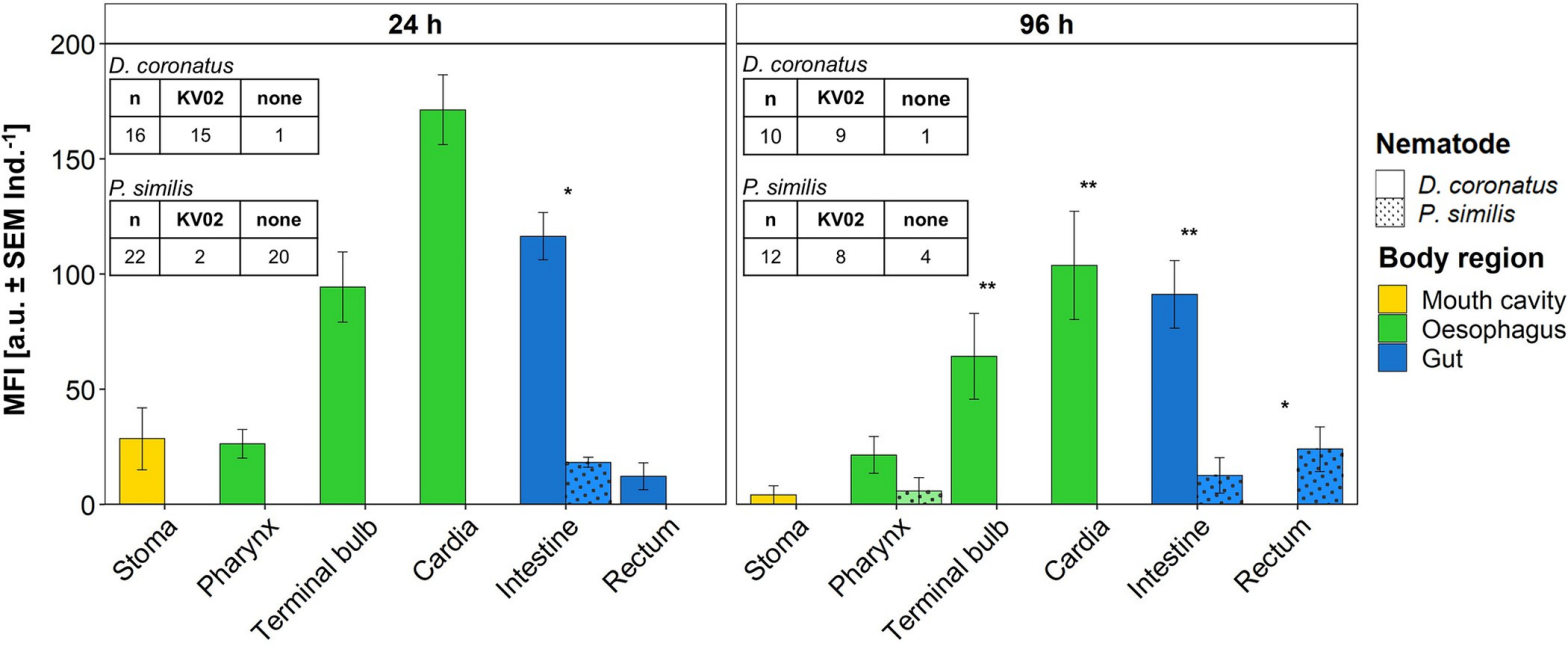
Presence of mCherry-labeled *Legionella pneumophila* KV02 in the digestive system of nematodes. *Diploscapter coronatus* (plain) and *Plectus similis* (dots) were incubated together in the same biofilm microcosm for 24 h and 96 h. Number of investigated individuals (n), number of individuals that harbored *L*. *pneumophila* KV02 (KV02) and number of individuals without bacteria are shown for each nematode species. The bacterial load is measured as mean fluorescence intensity (MFI: mean ± SEM Ind.^-1^). Statistical differences according to the Mann-Whitney U test with * and ** at p < 0.05 and p < 0.01, respectively.

To test whether interspecific competition of bacterial feeders affects the uptake of *L*. *pneumophila* KV02, the nematodes *D*. *coronatus* and *P*. *similis* were incubated in the same microcosms. After 24 h incubation, the bacterial load of the intestine was significantly higher in *D*. *coronatus* (116 a.u. Ind^-1^) than in *P*. *similis* (18 a.u. Ind^-1^; p < 0.015) (Fig 3). A similar species-specific bacterial ingestion rate was observed after 96 h, with 91 and 13 a.u. Ind^-1^ for *D*. *coronatus* and *P*. *similis*, respectively (p < 0.021). After 24 h, the bacterial load of the intestine did not differ to the respective single-species set-ups. On the other hand, after 96 h in the mixed-species set-up, *D*. *coronatus* and *P*. *similis* harbored significantly more bacteria than in the respective single-species set-ups (*D*. *coronatus* = p < 0.021; *P*. *similis* = p < 0.012). Further, for *D*. *coronatus*, also the bacterial load of the pharynx with 22 a.u. Ind^-1^ (p < 0.001) and cardia with 104 a.u. Ind^-1^ (p < 0.044) was higher in the mixed compared to the single set-up.

Regarding the other body compartments of nematodes, the overall fluorescence pattern in *D*. *coronatus* in mixed- and single-species set-up did not differ. The terminal bulb, cardia and intestine had the highest bacteria load after 24 h (87% of individuals, n = 16), as well as after 96 h incubation (80% of individuals, n = 10). Similarly, for *P*. *similis*, the overall fluorescence pattern in mixed- and single-species set-up did not differ. After 96 h incubation in the mixed-species set-up, bacteria were distributed between pharynx, intestine and rectum with a mean of 67% of nematodes observed (n = 12).

### 3.2 Bacterial choice assays—*L*. *pneumophila* versus *E*. *coli*

#### Single-species set-up

**Fig 4:**
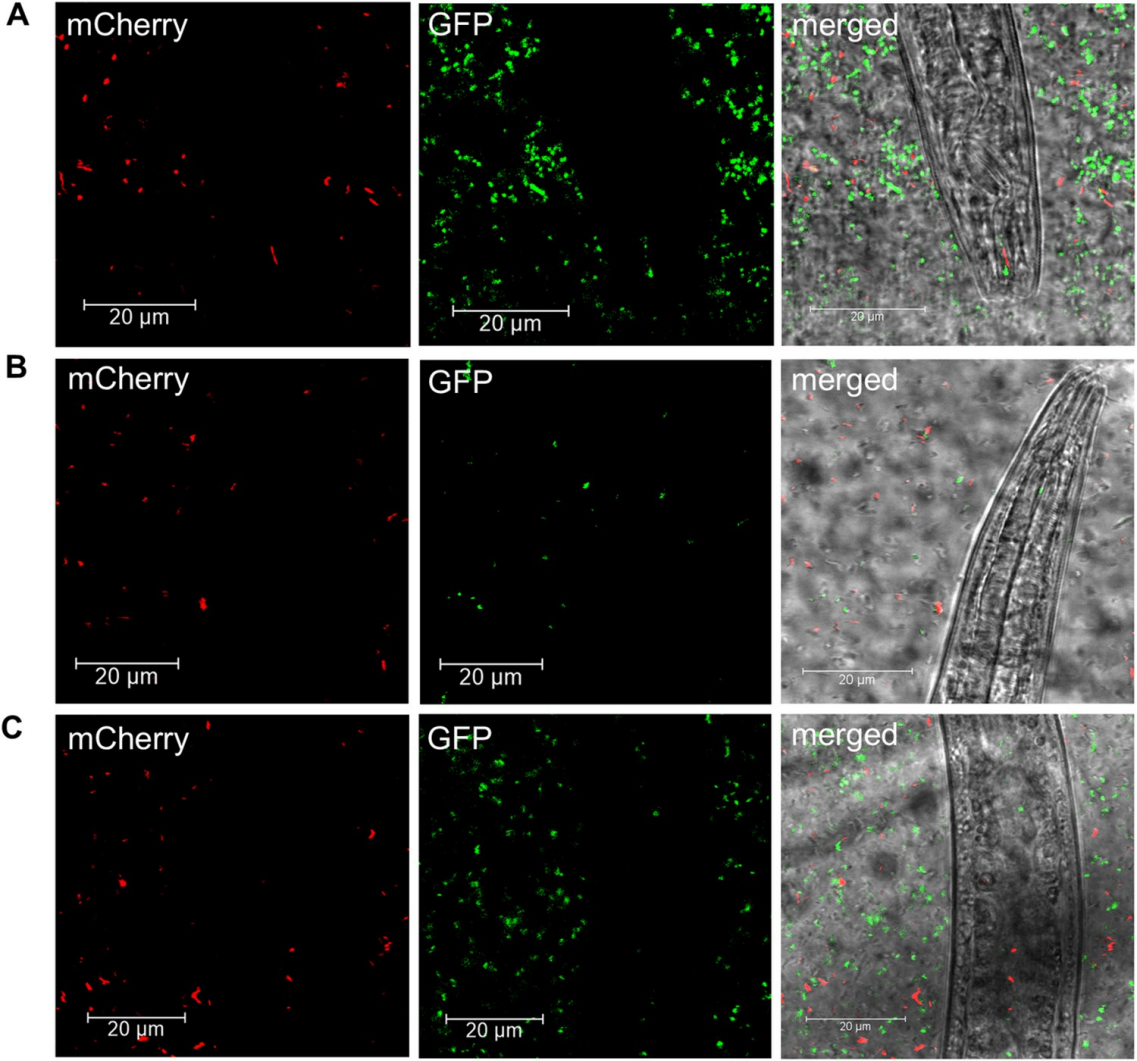
*Legionella pneumophila* KV02 and *Escherichia coli* DH10β in the digestive system of *Plectus similis*. *L*. *pneumophila* KV02 (mCherry, red), *E*. *coli* DH10β (GFP, green) and *P*. *similis* were incubated together for 24 h in a biofilm model. (A) Stoma. (B) Pharynx. (C) Intestine. Confocal laser scanning microscopy images show the distribution of *L*. *pneumophila* KV02 (left) and *E*. *coli* DH10β (middle) separately and in merged images (right).

**Fig 5:**
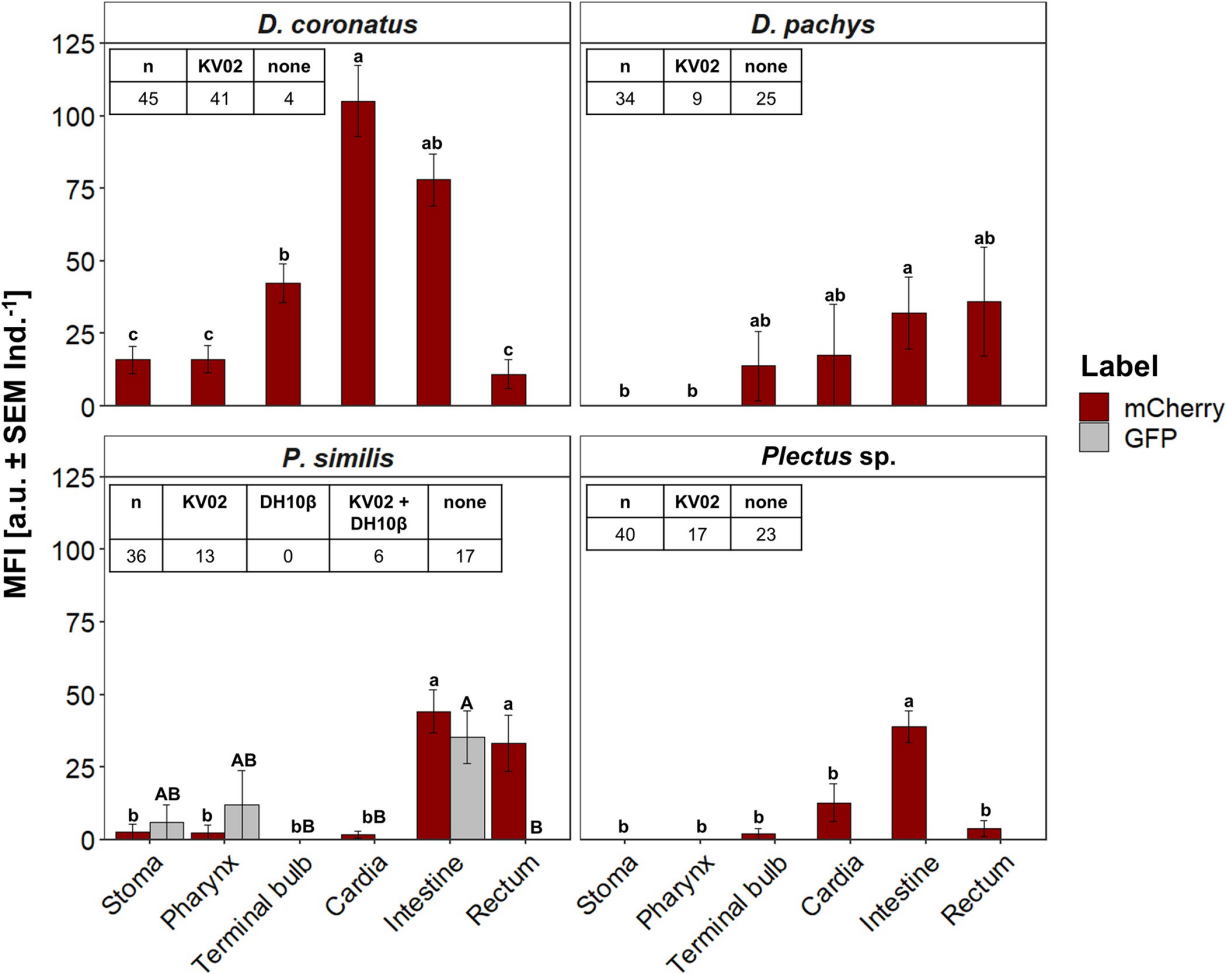
Presence of mCherry-labeled *Legionella pneumophila* KV02 and GFP-labeled *Escherichia coli* DH10β in nematodes. *L*. *pneumophila* KV02 (red), *E*. *coli* DH10β (gray) and nematodes were incubated together for 24 h in a biofilm model. Number of investigated individuals (n), number of individuals that harbored *L*. *pneumophila* KV02 (KV02) or *E*. *coli* DH10β (DH10β) and number of individuals without bacteria are shown for each nematode species. The bacterial load is measured as mean fluorescence intensity (MFI: mean ± SEM Ind^.-1^). Bars with no or the same letters are not statistically different according to Dunn’s post hoc test (p < 0.05).

To investigate potential food preference when given a choice between the human pathogen and a standard bacterial diet, nematodes were incubated with biofilm inoculated simultaneously with *L*. *pneumophila* KV02 and *E*. *coli* DH10β. After 24 h, *P*. *similis* was the only nematode species feeding on both bacteria (Fig 4). In 17% of the 36 specimens investigated high abundance occurred in the intestine with 35 and 44 a.u. Ind^-1^ for *E*. *coli* DH10β and *L*. *pneumophila* KV02, respectively (Fig 5). Fluorescence signals were also detected in the stoma and pharynx with MFIs more than twice as high for *E*. *coli* DH10β. On the other hand, solely *L*. *pneumophila* KV02 was present around the cardia (2 a.u. Ind^-1^) and in the rectum (33 a.u. Ind^-1^) of *P*. *similis*, where *E*. *coli* DH10β was completely lacking.

The other three nematode species tested did only feed on *L*. *pneumophila* KV02, and no cells of *E*. *coli* DH10β were detected after 24 h. This was surprising, as general palatability and ingestion of *E*. *coli* DH10β were verified in a control set-up (S3 Fig). The species exhibited different fluorescence patterns (Fig 5): The overall MFI of *L*. *pneumophila* KV02 was significantly higher in *D*. *coronatus* compared to *Plectus* sp. and *D*. *pachys* (p < 0.001). Fluorescence was detected in more than 90% of the *D*. *coronatus* specimen, but only in 43 and 26% of the observed *Plectus* sp. and *D*. *pachys*, respectively. In detail, for *D*. *coronatus* the highest bacterial load occurred around the cardia (105 a.u. Ind^-1^), followed by the intestine (78 a.u. Ind^-1^), while less bacteria were detected in the stoma, pharynx and rectum (p < 0.001). In *D*. *pachys*, the bacterial load increased from 14 a.u. Ind^-1^in the terminal bulb over 32 a.u. Ind^-1^ in the intestine to 36 a.u. Ind^-1^ in the rectum. No fluorescence was detected in the stoma and pharynx. In *Plectus* sp., the bacterial load was highest in the intestine (39 a.u. Ind^-1^) and significantly lower in all other body compartments (p < 0.001).

After 96 h incubation none of the four nematode species contained *E*. *coli* DH10β (S4 Fig), while cells of *L*. *pneumophila* KV02 were still evident. The overall fluorescence pattern of the bacteria in the nematodes were not affected by time, with signals comparable to 24 h, i.e. again highest around the cardia (*D*. *coronatus*) or intestine (*D*. *pachys*, *P*. *similis*, *Plectus* sp.).

#### Mixed-species set-up

**Fig 6:**
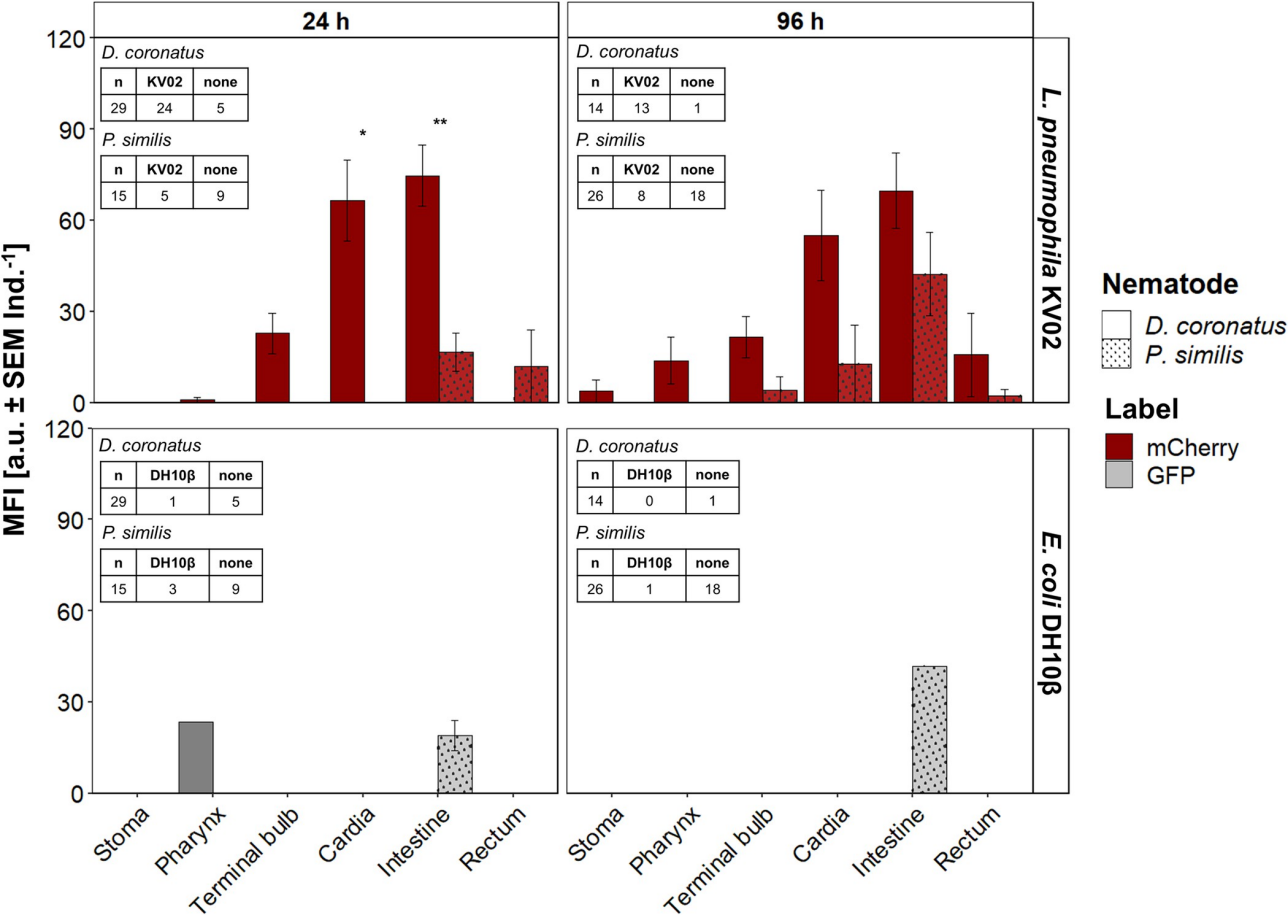
Presence of mCherry-labeled *Legionella pneumophila* KV02 and GFP-labeled *E*. *coli* DH10β in nematodes. *L*. *pneumophila* KV02 (red), *E*. *coli* DH10β (gray) and the nematode species *Diploscapter coronatus* (plain) and *Plectus similis* (dots) were incubated together in the same biofilm microcosm for 24 h and 96 h. Number of investigated individuals (n), number of individuals that harbored *L*. *pneumophila* KV02 (KV02) or *E*. *coli* DH10β (DH10β) and number of individuals without bacteria are shown for each nematode species. The bacterial load is measured as mean fluorescence intensity (MFI: mean ± SEM Ind.^-1^). Statistical differences according to the Mann-Whitney U test with * and ** at p < 0.05 and p < 0.01, respectively.

Incubation of different nematode species in the same biofilm had variable effects on the ingestion and distribution of *L*. *pneumophila* KV02 and *E*. *coli* DH10β in their digestive system (Fig 6). Generally, the bacterial load of *L*. *pneumophila* KV02 was always higher in *D*. *coronatus* than *P*. *similis* (except in the rectum after 24 h incubation), which was significant for the cardia (p < 0.012) and intestine (p < 0.002) after 24 h incubation. Moreover, irrespective of the presence of another bacterial feeder, in *D*. *coronatus* the cardia and intestine showed the highest bacterial loads, and *P*. *similis* harbored most *L*. *pneumophila* KV02 in its intestine.

Comparable to the single-species set-ups, *E*. *coli* DH10β scarcely was detected in *D*. *coronatus*, with only one individual exhibiting a fluorescence signal in the pharynx after 24 h incubation (24 a.u. Ind^-1^). In contrast, the bacterial location in *P*. *similis* varied. While in single species set-ups, nematodes harbored *E*. *coli* DH10β in stoma, pharynx and intestine, in the presence of *D*. *coronatus* bacteria were only detected in the intestine. Here, 19 and 42 a.u.

Ind^-1^ were detected after 24 and 96 h, respectively. This corresponds to a reduction in fluorescence by 46% after 24 h compared to the single-species set-up, yet this effect was not significant.

## 4. Discussion

### 4.1 Nematode traits affecting pathogen load

The co-occurrence of the human pathogen *L*. *pneumophila* and nematodes in cooling tower biofilms suggests nematodes as potential reservoir for the human pathogen. This is supported by the observation, that each tested nematode species readily fed on *L*. *pneumophila* KV02 in a semi-natural biofilm model. However, the bacterial load varied with genus as well as with species, as indicated by the fluorescence intensity of the ingested bacterial cells. In general, the number of ingested particles is dependent on feeding type (i.e. size and morphology of the stoma) and feeding behavior of nematodes [49, 50]. *Diploscapter*, as an enrichment opportunist (c-p 1) ingests food at high pumping rates of the pharynx, while *Plectus* (c-p 2) pumps more irregularly with active sweeping motions during feeding [51]. In line with this, *Diploscapter* had a higher mean load of *L*. *pneumophila* KV02 cells than *Plectus* in the single set-ups of both, the no-choice (*L*. *pneumophila*-only) and choice (*L*. *pneumophila* vs. *E*. *coli*) assays. Additionally, the wider stoma of *Diploscapter* (3.1 ± 0.5 μm) compared to *Plectus* (2.1 ± 0.3 μm) likely also contributed to its higher bacterial ingestion rates.

Differences in pharyngeal muscle contraction patterns between *Diploscapter* and *Plectus* may have also determined the bacterial loads as pumping behavior strongly affects the efficiency of pharyngeal food transport [52]. Further, *Plectus* has a less complex pharynx than *Diploscapter*, lacking a muscular median bulb, probably resulting in less efficient bacterial ingestion. For example, compared to *Poikilolaimus oxycercus* with median and terminal bulb, *Panagrolaimus* lacking the median bulb needs two more pumping cycles to transport particles towards the terminal bulb [53]. A morphological peculiarity of *Diploscapter* is the existence of two cuticularized chambers posterior the grinder. This so-called double haustrulum allows for a “rumination” of particles, as they can re-enter the grinder from the intestine. This increases the grinding efficiency as not all bacteria are crushed by the grinder in the first passage [53]. Grinding efficiency may therefore be a further factor influencing the bacterial load in the intestine of the tested genera.

Apart from uptake, the bacterial load in the intestine is determined by its length and volume, the absorption rate of digested products, and the defecation rate [54]. Likely, differences in the intestinal volume of *Diploscapter* and *Plectus* determined the number of bacterial cells entering the intestine, while the microvilli frequency and density of necessary transporters affected bacterial digestion [54], resulting in the observed differences in fluorescence intensities. The attachment of *L*. *pneumophila* to intestinal cells may be facilitated by the surface-associated heat shock protein Hsp60 [33]. While speculative, it may be possible that taxon-specific variations in membrane receptors affect the binding of Hsp60 to the intestinal cells of *Diploscapter* and *Plectus*.

Finally, defecation rates vary with nematode species [55], resulting in different bacterial loads. Schiemer et al. [56] reported that the assimilation efficiency, i.e. the ratio between ingestion and egestion (defecation) rate, in *Plectus palustris* decreases with increasing food availability as food particles pass the gut too quickly for effective digestion.

Overall, single intact *L*. *pneumophila* KV02 cells could be detected past the grinder in all tested nematode species. Subsequent defecation disperses undigested bacterial cells into the environment. Thus, in addition to *C*. *elegans*, the two investigated nematode taxa fulfill a major prerequisite to act as a reservoir and transmission vehicle for *L*. *pneumophila*.

### 4.2 Distribution pattern of *L*. *pneumophila* in the digestive tract of nematodes

The distribution pattern of *L*. *pneumophila* KV02 varied in the digestive tract of biofilm-dwelling nematodes. A key process of nematode feeding is the quick transport of food particles through the oesophagus into the intestine, which is realized by a continuous pumping motion of the pharynx. With a duration of ca. 300 ms per contraction-relaxation cycle [27, 57], pumping is too fast to effectively accumulate bacteria in the pharyngeal lumen. Instead, food particles are trapped in the terminal bulb of the oesophagus, before being transported into the intestine via the cardia. A corresponding fluorescence pattern, i.e. high fluorescence intensities around the cardia and further on in the intestine, but low intensities in stoma and pharynx, was best observable in *D*. *coronatus* (Fig 2). In contrast, in *D*. *pachys*, *P*. *similis* and *Plectus* sp. bacteria were quite evenly distributed across body regions.

In the current study, the bacterial load of the gut did not decrease with observation time, rather bacteria accumulated around the cardia and along the intestine at least for 96 h. As bacterial residence time in the intestine is regarded as short with 2 to 10 minutes [58], this indicates successful pathogen persistence despite the hosts immune response. Especially in *D*. *pachys* the evenly distributed *L*. *pneumophila* KV02 cells after 96 h incubation (Fig 2) may point to a *Legionella* parasitism. Yet, also commensalism might be a possible interaction, as no typical symptoms of *Legionella* infection, i.e. extrusion of viscera through the vulva and intestinal and anal distension [32] were detectable after 96 h of incubation. The bacterial load neither decreased nor increased with time, thus it cannot be concluded whether nematodes just harbored *L*. *pneumophila* KV02 or if bacterial multiplication took place. Yet, the presence of *L*. *pneumophila* KV02 in the nematode rectum at both observation times suggests that a fraction of cells is constantly released into the environment after the passage through the gut. As nematodes defecate 30 to 60% of ingested bacteria in viable conditions [59], this makes them potential transport vehicles of *Legionella* in cooling towers.

Overall, bacterial cells were not transported along the intestine immediately after leaving the oesophagus. This lag time in the cardia region as well as the intestine may offer the potential as replicative niche for human pathogens.

### 4.3 Nematode food choice and competition

To account for differences in nematode feeding traits as well as for interspecific competition multi-species set-ups with *D*. *coronatus* and *P*. *similis* in the same biofilm were conducted. Like in the single set-ups, the *L*. *pneumophila* KV02 load was clearly higher in the intestine of *D*. *coronatus* than *P*. *similis* at both observation times. Interestingly, the *L*. *pneumophila* KV02 fluorescence intensity in the *P*. *similis* intestine was significantly higher in single species set-ups than in the multi-species set-ups. This was the case for both the no-choice assay (after 96 h) and choice assay (after 24 h). In addition to species-specific morphological and metabolic traits (see above) this could be due to exploitation competition, i.e. *D*. *coronatus* and *P*. *similis* interacted indirectly as they competed for food. *D*. *coronatus*, as superior competitor, ingested *L*. *pneumophila* KV02 more rapidly thereby decreasing the amount of available food for *P*. *similis*.

Interestingly, in *D*. *coronatus*, *D*. *pachys* and *Plectus* sp. only the presence of *L*. *pneumophila* KV02 but not the presence of *E*. *coli* DH10β could be detected in the digestive tract when bacteria were offered simultaneously (Fig 5 and S4 Fig), which points to a food preference for the pathogen. *P*. *similis* was the only species feeding on both, *L*. *pneumophila* KV02 and *E*. *coli* DH10β after co-inoculation for 24 h (Figs 4 and 5). Perhaps, this result is related to a greater food requirement of *P*. *similis*, since it was the largest of the four tested species. For example, the species *P*. *palustris* consumes 7.2 × 10^6^ bacterial cells daily [60], while the smaller *Cephalobus persegnis* has a consumption rate of 6.6 × 10^5^ cells per day [61]. Thus, it may be possible that when the density of the preferred diet *L*. *pneumophila* KV02 was declining, *P*. *similis* started feeding on the less preferred *E*. *coli* DH10β, too.

In contrast to the biofilm model, a previous study showed that, compared to *E*. *coli* OP50, *L*. *pneumophila* KV02 significantly impairs the pharyngeal pumping activity, i.e. pumping rates were reduced by 70% in *P*. *similis* and even by 300% in *Plectus* sp. on agar plate [36]. Apparently, the semi-natural test environment of a biofilm results in a distinctly different nematode food choice compared to agar plate assays. This fact emphases the need for experiments that reflect the habitat conditions in order to achieve realistic interactions between bacteria and nematodes.

Several reasons may explain the differences detected between agar plate and biofilm assays. Firstly, the co-ingestion of the natural biofilm microbiota could have altered the pathogenicity of *L*. *pneumophila*. Indeed, feeding co-cultures of beneficial and detrimental bacteria to *C*. *elegans* resulted in the active suppression of the pathogenicity of certain detrimental strains [62]. Moreover, *Bacillus megaterium* and *Pseudomonas mendocina* increased resistance to infection with *P*. *aeruginosa* in *C*. *elegans*, compared to individuals fed with *E*. *coli* OP50 [63]. Also, nematode exposure to pathogenic agents varies between agar plate and natural habitats. While the aquatic biofilm environment likely has attenuated the impact of effector proteins secreted by *Legionella*, these were not diluted on agar plate, thereby downregulating the grazing activity of nematodes. Secondly, nematodes can develop strategies to avoid potential pathogens, called aversive learning, i.e. nematodes modify their food preference after pathogen infection and cellular damage [64]. For instance, the initial preference of *C*. *elegans* for *P*. *aeruginosa* PA14 over *E*. *coli* OP50 was reversed after feeding on the pathogen, ultimately resulting in a preference for *E*. *coli* [65]. However, no diet switch from *L*. *pneumophila* to *E*. *coli* was observed over the entire experimental period, suggesting aversive learning as unlikely. Thirdly, a crucial process for bacterial biofilm formation is known as quorum sensing mediated by extracellular signal molecules (autoinducers—AIs) for cell-cell communication [66]. Several AIs are recognized by nematodes and stimulate chemotaxis and feeding, including the acylated homoserine lactone (aHSL) AIs of Gram-negative bacteria [67]. Thus, *Legionella* AIs functioning as chemoattractant for nematodes might explain the preference for *L*. *pneumophila* KV02 over *E*. *coli* DH10β in the biofilm assays.

Shaheen & Ashbolt [68] investigated the feeding preferences of free-living amoebae regarding *L*. *pneumophila* and two *E*. *coli* strains in amoeba-bacteria co-cultures. Using fluorescence microscopy, they observed a “forced-feeding” situation, where amoebae mostly fed on *L*. *pneumophila*, when the preferred *E*. *coli* sources were depleted [68]. However, no remarkable decrease of *E*. *coli* DH10β cells could be determined in the biofilm microcosms after incubation with nematodes for 24 h. Also, the existence of the natural biofilm microbial community as an alternative food source to *L*. *pneumophila* KV02 speaks against such a "forced-feeding" condition.

Another aspect is competition for colonization niches in the nematode gut between *L*. *pneumophila* KV02 and *E*. *coli* DH10β. The higher *Legionella* load may indicate that the pathogen has a competitive advantage against non-pathogenic strains, i.e. *E*. *coli* DH10β has difficulties surviving in an environment in which an active host response, triggered by *Legionella*, is present. This immune system response includes pathways involving e.g. the DAF-2 (insulin/IGF-I like) receptor, p38 MAP kinase and the transforming growth factor β (TGF-β) [43]. For instance, *C*. *elegans* p38 MAP kinase mutants were more, while *daf-2* insulin signaling pathways mutants were less susceptible to *Legionella* infection [32]. Indeed, Portal-Celhay & Blaser [69] reported that in *C*. *elegans* grown on mixed lawns of bacteria, *S*. *enterica* is able to establish persistent colonization in the gut, while completely outcompeting *E*. *coli* OP50. Yet, *S*. *enterica* also outcompeted *E*. *coli* OP50 in *daf-2* mutants, which indicates that gut colonization resilience is more bacterial strain specific and less dependent on innate immunity [69].

In sum, all tested nematode species exhibited a high load of *L*. *pneumophila* KV02 but a low presence of *E*. *coli* DH10β in their gut (except *P*. *similis*), when feeding in the semi-natural biofilm environment. This indicates a preference for the pathogen over the non-fluorescent autochthonous microflora and GFP-labeled *E*. *coli* DH10β. Competition between the bacterial strains within the nematode gut may have reduced the presence of *E*. *coli* DH10β additionally. The underlying mechanisms of *Legionella*-nematode interactions have to be further explored in test systems reflecting natural habitat conditions.

## 5. Conclusion

This study using a semi-natural biofilm model provides first evidence that bacterial-feeding nematodes co-occurring with *L*. *pneumophila* in cooling towers preferentially ingest the human pathogen over the standard laboratory diet *E*. *coli*. The comparison between bacterial load patterns in *Diploscapter* and *Plectus* revealed species-specific regions for bacterial accumulation, which may have implications for their potential as a replicative niche for *L*. *pneumophila*. A high load of *L*. *pneumophila* compared to common bacteria such as *E*. *coli* may be a sign of food preference, yet bacterial competition within the nematode gut cannot be excluded. The presence of the pathogen after 96 h underlines the potential of biofilm-dwelling nematodes to serve as reservoir and transmission vehicle of *L*. *pneumophila* in cooling towers. Future environmental studies on nematode-*Legionella* interactions should consider a wider time span to reveal possible long-term effects of *Legionella* pathogenicity on nematode grazing. Additionally, investigation of different experimental temperatures is desirable to mimic alterations in the thermal regime of cooling towers.

## Supporting information

S1 Fig Illustration of the experimental set-up. (A) Subsample of the cooling tower biofilm material. (B) Illustration of a chamber slide. 250 μl of nematode-free biofilm within separate chambers as experimental microcosms. Depending on the assay, either 50 μl of mCherry-labeled *L*. *pneumophila* KV02 (2 x 10^8^ CFU ml^-1^), or 50 μl of GFP-labeled *E*. *coli* DH10β together with 50 μl of mCherry-labeled *L*. *pneumophila* KV02 (6 x 10^8^ CFU ml^-1^), resuspended in sterile-filtered cooling tower water, were inoculated and left overnight at 25°C to adapt. (C) Nematode inoculation. Hand-picked nematodes (30 individuals per microcosm) were inoculated. Two scenarios were investigated: single set-ups each comprised one of the four test species (*D*. *coronatus*, *D*. *pachys*, *P*. *similis*, *Plectus* sp.), while mixed set-ups were a combination of *P*. *similis* and *D*. *coronatus* with each 15 individuals, respectively, per microcosm (n = 2). All microcosms were incubated at 25°C for either 24 h or 96 h.
(TIF)

S2 Fig Choice index (CI) after Abada et al. [44]. The GFP-labeled strain *E*. *coli* DH10β was tested against the isogenic strain DH10β not expressing GFP, as well as against *E*. *coli* OP50. A CI of -1.0 represents a total preference for the GFP-expressing strain, a CI of 1.0 represents a total preference for the isogenic strains DH10β or OP50, while a CI of 0 represents an equal distribution of nematode individuals between both bacterial diets. Food choice assays were performed on 9 cm NGM agar plates at 20°C. For this, 30 μl of freshly prepared bacterial suspensions adjusted to 10^6^ CFU ml^-1^ were seeded at opposite sides of the assay plate at a distance of 1.5 cm from the plates’ edge. Then, 100 young adult nematodes suspended in sterile mineral water (Volvic, Danone Deutschland GmbH, Germany) were placed at the center of each plate. Each trial was replicated 5 times with 3 repeats per replicate. Bars with no or the same letters are not statistically different according to the Tukey HSD test (p < 0.05).
(TIF)

S3 Fig Representative confocal laser scanning microscopy images of nematodes harboring *E*. *coli* DH10β. Nematodes harbor *E*. *coli* DH10β (GFP, green) in the pharynx (*Diploscapter coronatus*, *Plectus* sp.), terminal bulb (*Plectus similis*), and rectum (*Diploscapter pachys*). Bacteria and nematodes were incubated in sterile-filtered cooling tower water for 24 h at 25°C.
(TIF)

S4 Fig Presence of mCherry-labeled *Legionella pneumophila* KV02 and GFP-labeled *Escherichia coli* DH10β in nematodes. *L*. *pneumophila* KV02 (red), *E*. *coli* DH10β (gray) and nematodes were incubated together for 96 h in a biofilm model. Number of investigated individuals (n), number of individuals that harbored *L*. *pneumophila* KV02 (KV02) and number of individuals without bacteria are shown for each nematode species. The bacterial load is measured as mean fluorescence intensity (MFI: mean ± SEM Ind^.-1^). Bars with no or the same letters are not statistically different according to Dunn’s post hoc test (p < 0.05).
(TIF)

S1 Dataset Choice index of nematodes. (XLSX)

S2 Dataset Mean fluorescence intensity (MFI) of *Legionella pneumophila* (no-choice assay). (XLSX)

S3 Dataset Mean fluorescence intensity (MFI) of *Legionella pneumophila* and *Escherichia coli* DH10β (choice assay). (XLSX)
