# Supplementary figures and images for: Biotic interactions between the human pathogen Legionella pneumophila and nematode grazers in cooling tower biofilms
Source: PLoS One. 2024 Oct 25;19(10):e0309820. doi: 10.1371/journal.pone.0309820 (PMC11508163; doi:10.1371/journal.pone.0309820)

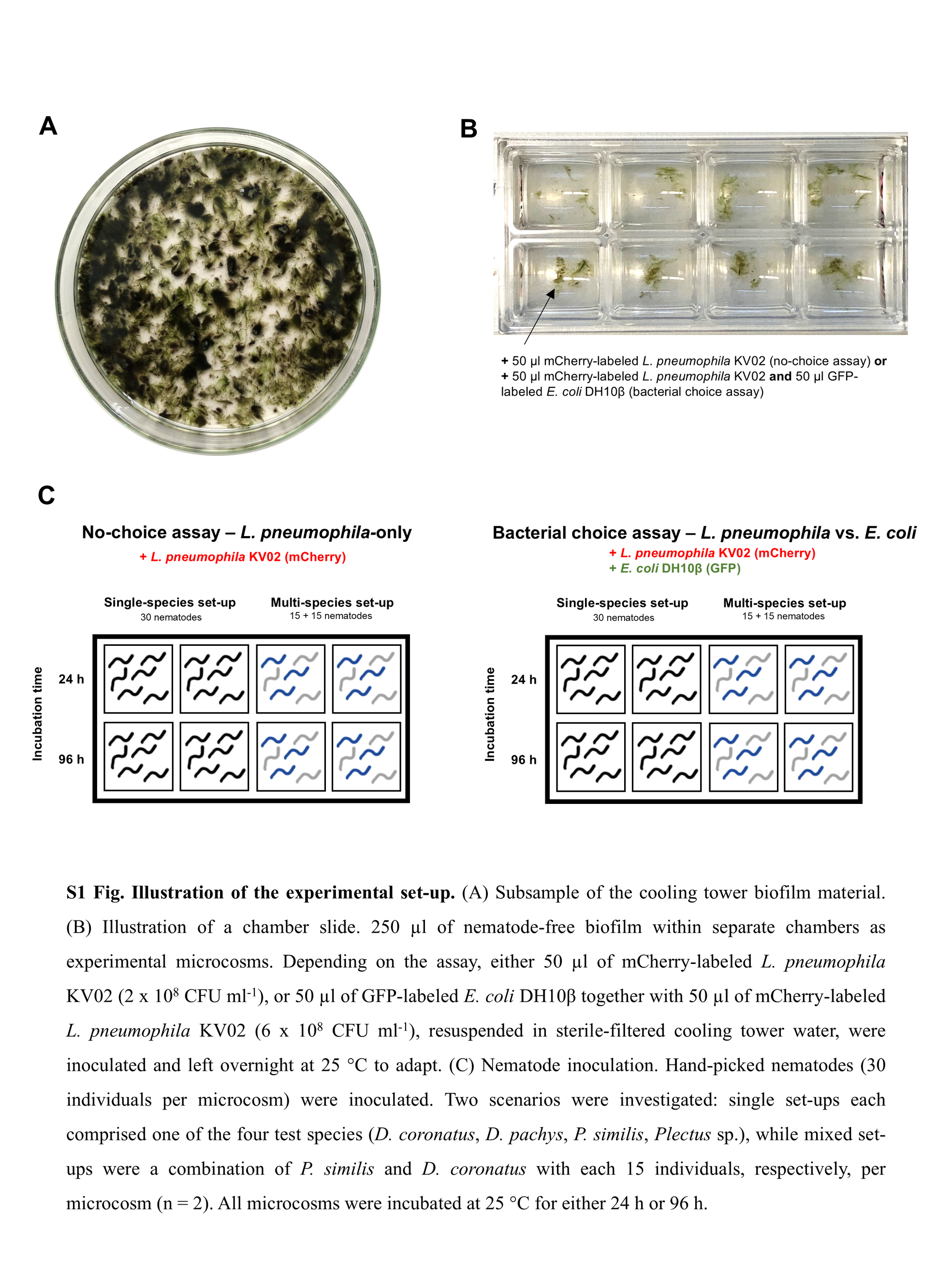

Supplement: S1 Fig — (A) Subsample of the cooling tower biofilm material. (B) Illustration of a chamber slide. 250 μl of nematode-free biofilm within separate chambers as experimental microcosms. Depending on the assay, either 50 μl of mCherry-labeled L. pneumophila KV02 (2 x 10^8^ CFU ml-1), or 50 μl of GFP-labeled E. coli DH10β together with 50 μl of mCherry-labeled L. pneumophila KV02 (6 x 10^8^ CFU ml-1), resuspended in sterile-filtered cooling tower water, were inoculated and left overnight at 25°C to adapt. (C) Nematode inoculation. Hand-picked nematodes (30 individuals per microcosm) were inoculated. Two scenarios were investigated: single set-ups each comprised one of the four test species (D. coronatus, D. pachys, P. similis, Plectus sp.), while mixed set-ups were a combination of P. similis and D. coronatus with each 15 individuals, respectively, per microcosm (n = 2). All microcosms were incubated at 25°C for either 24 h or 96 h. (TIF) [file pone.0309820.s001.tif]

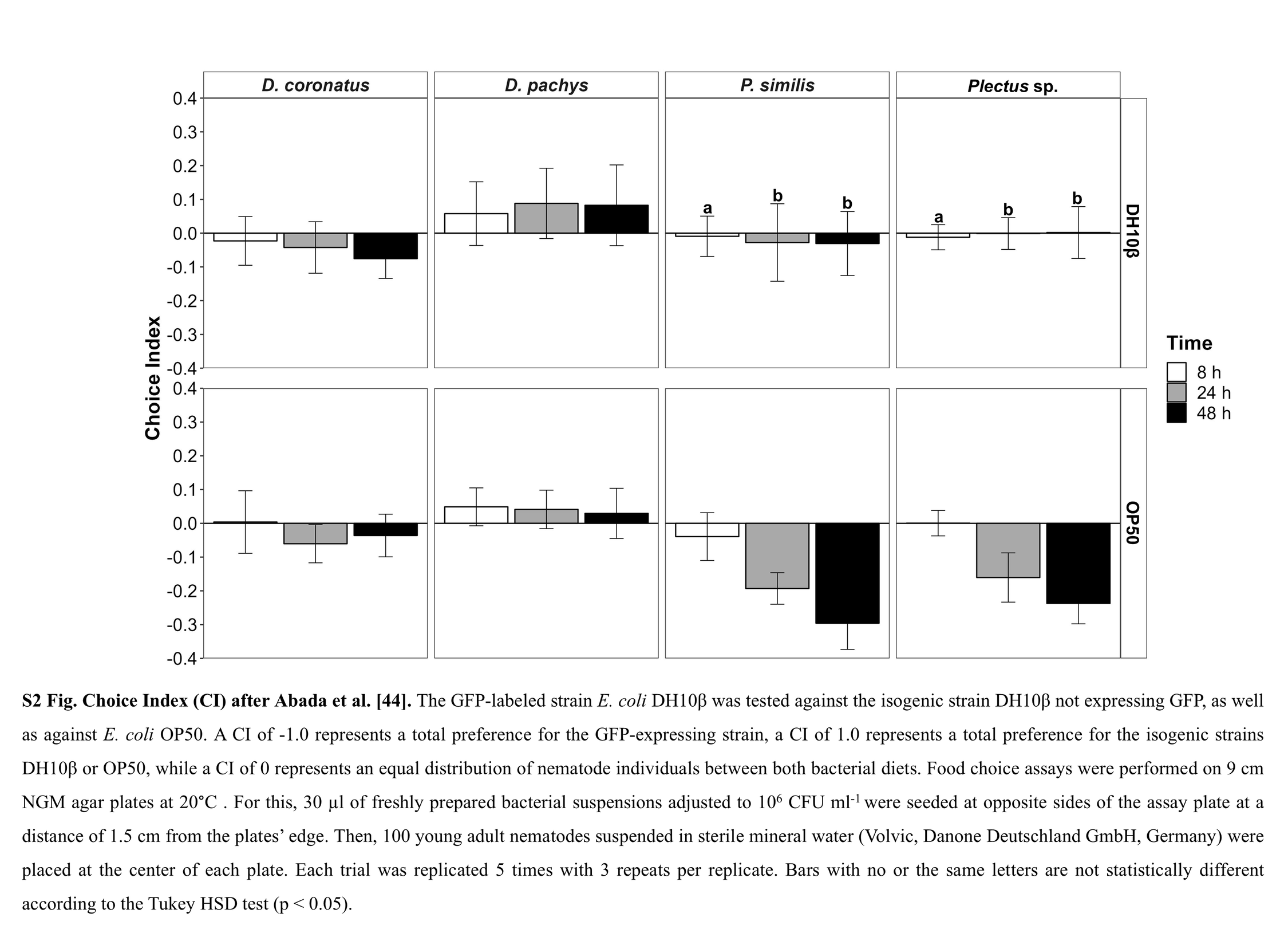

Supplement: S2 Fig — The GFP-labeled strain E. coli DH10β was tested against the isogenic strain DH10β not expressing GFP, as well as against E. coli OP50. A CI of -1.0 represents a total preference for the GFP-expressing strain, a CI of 1.0 represents a total preference for the isogenic strains DH10β or OP50, while a CI of 0 represents an equal distribution of nematode individuals between both bacterial diets. Food choice assays were performed on 9 cm NGM agar plates at 20°C. For this, 30 μl of freshly prepared bacterial suspensions adjusted to 106 CFU ml-1 were seeded at opposite sides of the assay plate at a distance of 1.5 cm from the plates’ edge. Then, 100 young adult nematodes suspended in sterile mineral water (Volvic, Danone Deutschland GmbH, Germany) were placed at the center of each plate. Each trial was replicated 5 times with 3 repeats per replicate. Bars with no or the same letters are not statistically different according to the Tukey HSD test (p < 0.05). (TIF) [file pone.0309820.s002.tif]

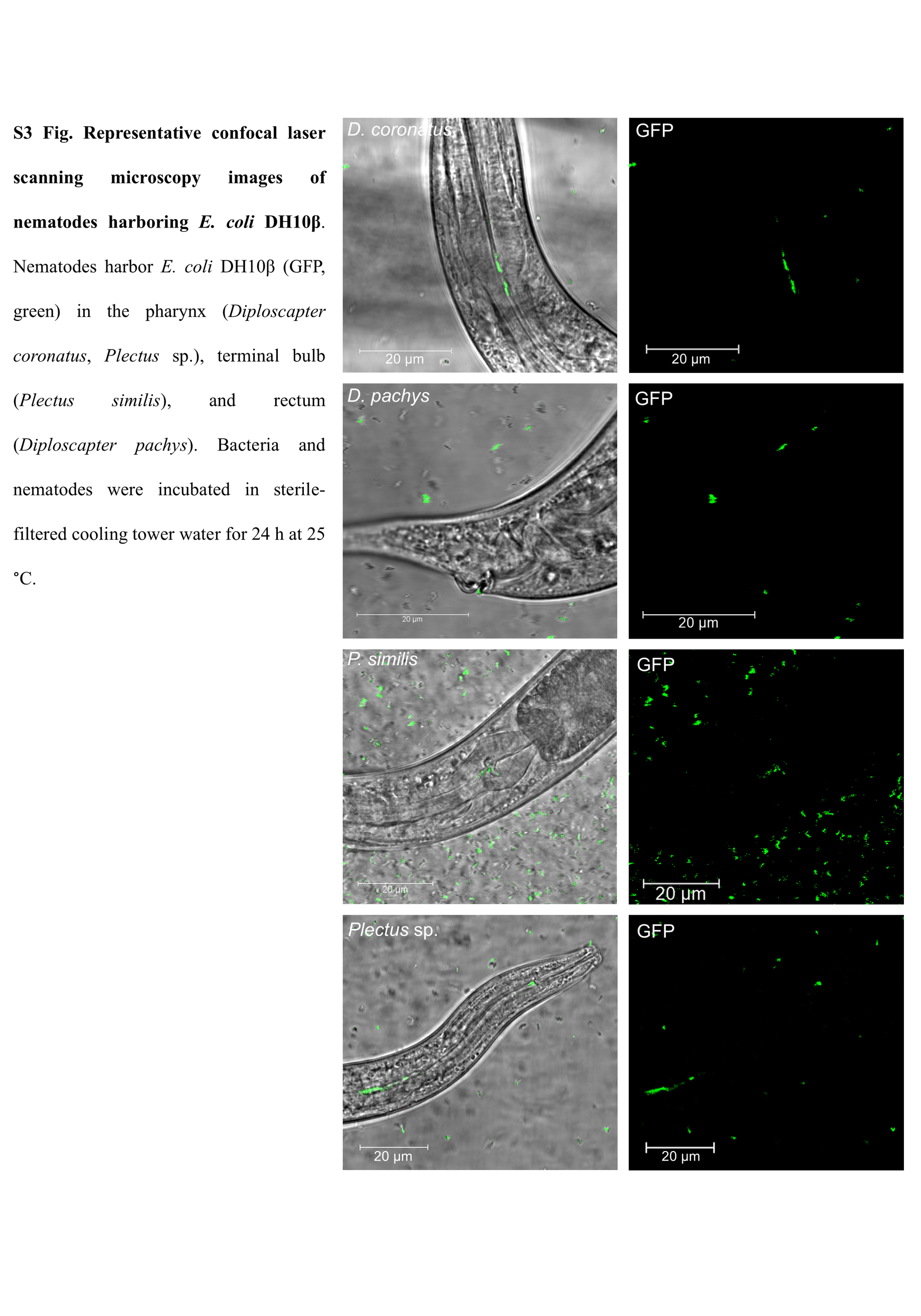

Supplement: S3 Fig — Nematodes harbor E. coli DH10β (GFP, green) in the pharynx (Diploscapter coronatus, Plectus sp.), terminal bulb (Plectus similis), and rectum (Diploscapter pachys). Bacteria and nematodes were incubated in sterile-filtered cooling tower water for 24 h at 25°C. (TIF) [file pone.0309820.s003.tif]

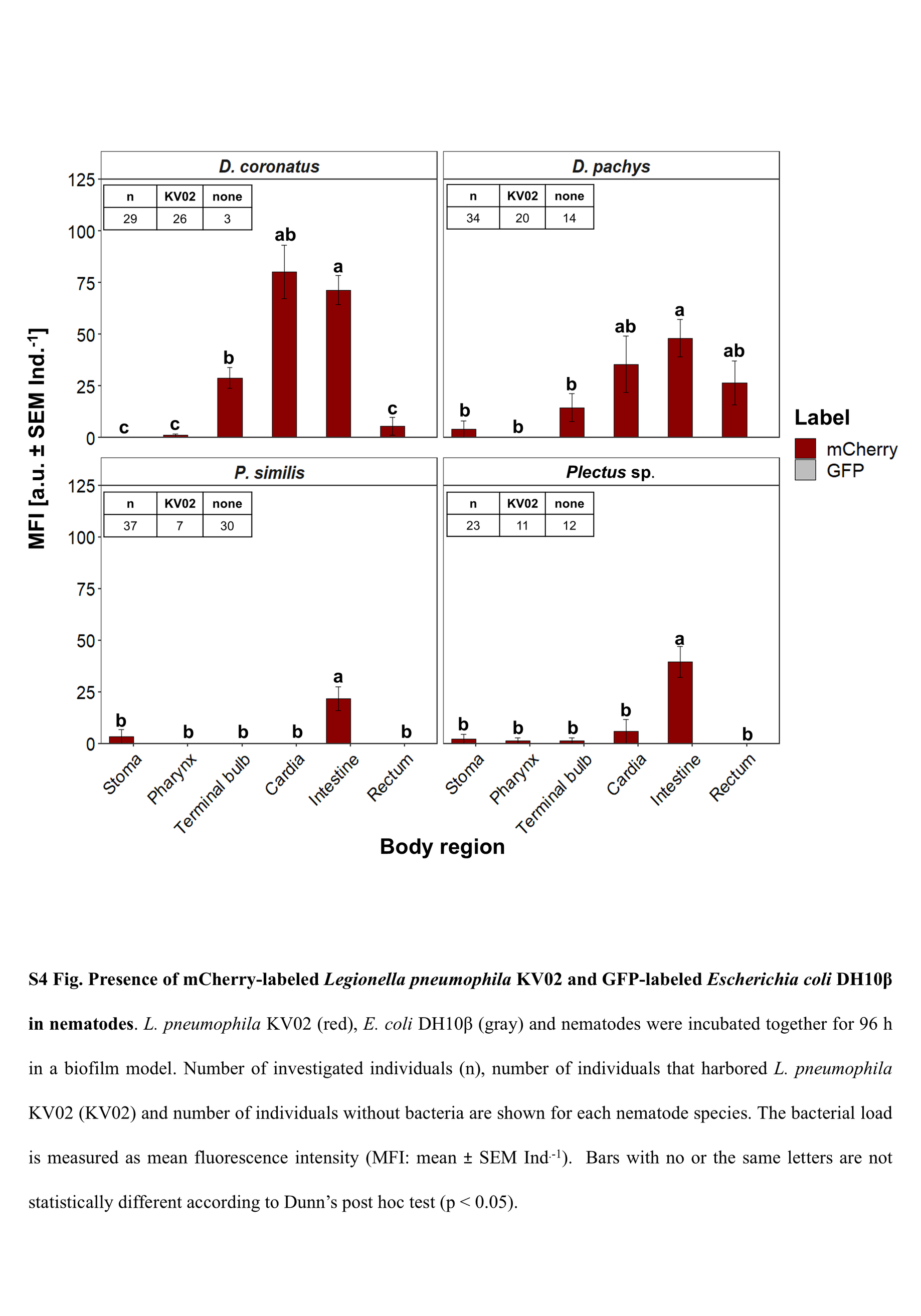

Supplement: S4 Fig — L. pneumophila KV02 (red), E. coli DH10β (gray) and nematodes were incubated together for 96 h in a biofilm model. Number of investigated individuals (n), number of individuals that harbored L. pneumophila KV02 (KV02) and number of individuals without bacteria are shown for each nematode species. The bacterial load is measured as mean fluorescence intensity (MFI: mean ± SEM Ind.-1). Bars with no or the same letters are not statistically different according to Dunn’s post hoc test (p < 0.05). (TIF) [file pone.0309820.s004.tif]
